# Emergence of a novel recombinant of CV-A5 in HFMD epidemics in Xiangyang, China

**DOI:** 10.1186/s12920-021-01107-6

**Published:** 2021-11-24

**Authors:** Yuting Yu, Zhiyu Luo, Weiping Jin, Jianyi Mai, Shasha Qian, Jia Lu, Zhenni Wei, Shengli Meng, Zejun Wang, Xuhua Guan, Yeqing Tong, Shuo Shen

**Affiliations:** 1 Wuhan Institute of Biological Products, Co. Ltd, Wuhan, 430207 People’s Republic of China; 2 Xiangyang Center for Disease Control and Prevention, Hubei, People’s Republic of China

**Keywords:** Coxsakievirus A5, Recombinantion, Hand, foot and mouse disease

## Abstract

**Background:**

Hand, foot and mouth disease (HFMD) is caused by a variety of enterovirus serotypes and the etiological spectrum worldwide has changed since a large scale of outbreaks occurred in 1997.

**Methods:**

A large number of clinical specimens of HFMD patients were collected in Xiangyang and genotyping was performed by qRT-PCR, conventional PCR amplification and sequencing. Among the 146 CV-A5 detected cases, the complete genome sequences of representative strains were determined for genotyping and for recombination analysis.

**Results:**

It was found that CV-A5 was one of the six major serotypes that caused the epidemic from October 2016 to December 2017. Phylogenetic analyses based on the VP1 sequences showed that these CV-A5 belonged to the genotype D which dominantly circulated in China. Recombination occurred between the CV-A5 and CV-A2 strains with a breakpoint in the 2A region at the nucleotide 3791.

**Conclusions:**

The result may explain the emergence of CV-A5 as one of the major pathogens of HFMD. A multivalent vaccine against HFMD is urgently needed to control the disease and to prevent emerging and spreading of new recombinants.

## Background

Hand, foot and mouth disease is caused by a variety of enteroviruses. At least more than 40 enterovirus serotypes were identified in epidemic and etiological surveillances from clinical samples of HFMD patients in mainland China and other countries [1–6]. The major serotypes currently prevailing in China are CV-A6, CV-A10, CV-A16, EV-A71 and other serotypes which either cause sporadic cases or small outbreaks [7]. HFMD is characterized by fever, sore throat, general malaise and vesicular eruptions on the hands, feet, oral mucosa and tongue. Severe cases include complications such as encephalitis, meningitis, acute flaccid paralysis, cardiorespiratory failure and even death. Historically, severe and fatal cases are mainly caused by EV-A71; however, in recent years, other serotypes are also responsible for the severity of HFMD cases [6].

Etiological spectrum of HFMD has been changing characteristically driven by the emergence of new predominant enterovirus serotypes such as CV-A6, CV-A10 and other serotypes. The driving forces of evolution are point mutations, intertypic and intratypic recombination [8]. CV-A5 previously caused sporadically HFMD, or herpangina, acute gastroenteritis and onychomadesis at low frequency or small enterovirus outbreaks worldwide [9–20]. Indeed, CV-A5 was recently associated with a relatively large proportion of HFMD cases in a largescale etiological investigation in Xiangyang performed in our laboratory. A total of 3, 703 case HFMD samples were collected and the typeable case number was 3201, which were genotyped by blasting and comparison with sequences of enteroviruses in GenBank. Of the typeable 3201 specimens, CV-A6 was the predominating serotype followed by CV-A16, CV-A10, CV-A5, CV-A2 and EV-A71 and the ratios were 51.47% (1906/3201), 15.3% (490/3201), 11.56% (370/3201), 4.56% (146/3201), 3.78% (121/3201) and 3.03% (97/3201), respectively. It seemed that these six serotypes were popular at the same time in Xiangyang [21].

CV-A5 is a member of species A in the genus *Enterovirus* within the family *Picornaviridae* in the order *Picornavirales* [22]. It has been further divided into clades A, B, C and D based on the divergence of the VP1 nucleotide sequence [23]. The single-stranded, polyadenylated, positive-sense RNA genome is proximately 7400 nucleotides in length. The genome contains an open reading frame (ORF) flanked by the untranslated regions (UTR) at both 5′- and 3′-terminals with a peptide VPg encoded by viral 3B protein, which covalently linked to the 5′ UTR. The polyprotein is co-translationally cleaved by viral proteases 2Apro, 3Cpro (or 3CDpro) into precursors such as P1, P2, P3, VP0, 3CD and mature structural proteins VP1-VP4 (1A-1D) and nonstructural proteins 2A-2C, 3A-3D.

In this study, the complete genome sequences of three RD cell isolates of CV-A5 were determined. These three isolates were the representative strains of 146 CV-A5 clinical specimens in an epidemic of HFMD outbreak in Xiangyang in 2017. Phylogenetic analysis revealed that a novel recombinant emerged carrying the 5′-terminal sequence of CV-A5 and the 3′-terminal sequence of CV-A2 with the recombination junction located at 2A region. The recombination may be responsible for the high proportion of CV-A5 HFMD cases among all enteroviruses detected. This novel recombinant may explain the outbreak caused by CV-A5 and emphasize the role played by recombination in the emerging, prevailing and changing of etiological spectrum of HFMD-associated enteroviruses.

## Materials and methods

### Collection of clinical data

Rectal swabs were collected from children with the symptoms of HFMD from three hospitals in Xiangyang, Hubei, China. The samples were transferred by viral transport medium Youkang (Beijing, China) containing Hanks balanced salt solution. The specimens were stored at -80 °C. The viral RNAs were detected by real time RT‑PCR and conventional PCR. All specimens were inoculated in monolayer of RD and Vero cells for virus isolation.

### Enterovirus isolation and RNA extraction

All of CV-A5 strains were sampled from April to July in 2017, among them CV-A5 R3474, CV-A5 R3487, CV-A5 R3490 were collected in April and isolated in RD cells and Vero cells by conventional methods [24], respectively. Samples were identified by molecular typing method. The viral RNAs were extracted directly from cell lysates.

### VP1 region sequencing and typing of CV-A5 strain

The VP1 regions were amplified by enterovirus VP1 universal primers (292 and 222) [25] listed in Table 1. The reaction was performed by adding 2 μl reaction buffer, 0.5 μl cDNA template, 3.7 μl enzyme mixture, 0.6 μl forward primer, 0.6 μl reverse primer (1.0 ng/μl each), and nuclease-free water to be a total volume of 20 μl. The PCR amplification was performed under the following conditions: 94 °C 5 min; 35 cycles of 94 °C 30 s, 55 °C 30 s, and 72 °C 30 s; 72 °C 10 min. VP1 sequences were compared with reference sequences available in GenBank. Virus strains which showed more than 75% nucleotide similarity with known enterovirus serotypes were considered to be the same serotype.

**Table 1:** Universal primers for Enterovirus VP1

Name	Primer sequence^a^ (5′–3′)	Position^b^ (nt)
292	5′-MIGCIGYIGARACNGG-3′	2612–2627
222	5′-CICCIGGIGGIAYRWACAT-3′	2969–2951

^a^IUB fuzzy code; I: deoxyinosine; M: A/C; Y, C/T; R: A/G; N: A/T/G/C; W: A/T

^b^The location relative to the genome of the poliovirus type I Mahoney strain

### Full-length genome sequencing

The complete genome of CV-A5 strains were sequenced for further genetic characterization. PCR conditions: 94 °C 5 min; 94 °C 30 s, 52 °C 30 s, 72 °C kb/30 s, 35 cycles; 72 °C 10 min. Primers used were listed in Table 2.

**Table 2:** Primers used for PCR amplification and sequencing of CV-A5 genome

Name	Primer sequence (5′–3′)	Position (nt)
A5 1F	TTAAAACAGCCTGTGGGTTGTACC	1–24
A5 6F	GCAATCCACTATGGTAGGACAACT	1998–2021
A5 9F	GCACCTTTTCAGTGAGATTCGTT	3128–3250
A5 9R	ACCTACTGCAAGCATGAGATG	3630–3610
A5 13R	AACCCAGCAAAGAGCTTGTAGATA	4825–4806
A5 20F	AAGAGAGTCTACGCCCTGGAG	4366–4386
A5 23R	TTGACCCCTTGTTCATCCACTAAT	5588–5565
A5 24F	TCACTGCTGAGAAGGAATATCAGG	5407–5430
A5 24R	CCAAAATGTATCTGGATTGCACCC	6585–6562
A5 polyA	AGTCAAGTTACATAGTAGGCTACAGTAACTGCTTTTTTTTTTTTTTTTTT	7370–7419

### Phylogenetic analysis and recombination analysis

The nucleotide homology among three CV-A5 strains from Xiangyang was 99.6%. The CV-A5 R3487 strain was selected to compare its nucleotide and amino acid sequence with those of other enterovirus by Megalign software. The phylogenetic tree was generated by MEGA-X software and the evolutionary distance was calculated with the neighbor-joining method using 500 boostraps for evaluation. The recombination was analyzed by using the MEGA-X and SimPolt software, through the Bootstaps method (500 Boostraps Replications) and the Kimura (2-parameter) model mapping, respectively. The Recombination Detection Program (RDP) was used for the detection of potential recombinant sequences and localization of possible recombination breakpoints.

### Nucleotide sequence accession number

16 CV-A5 strains VP1 nucleotide sequence downloaded from Genebank (888 nucleotides) and the full-length genome sequences of CV-A5 R3474, CV-A5 R3487, CV-A5 R3490 strains were determined in this study. The complete genome sequence of CV-A5 R3487, CV-A5 R3474, CV-A5 R3490 were deposited in GeneBank under the accession no MN663160, OK334537, OK334538.

## Results

### Detection and cell isolation of CV-A5

Among 4415 cases of clinically diagnosed HFMD, 3781 (85.64%) were confirmed by real time RT‑PCR and conventional PCR to be accounted for enteroviruses from Oct 2016 to Dec 2017 at Xiangyang. These included 78 cases of EV-A71 (1.77%) and 3703 cases of (83.87%) non-EV-A71 enteroviruses. Of these 3703 samples, serotyping and genotyping were performed by sequencing of amplified PCR fragments. The results showed that 86.44% of HEVs (3201/3703) were typeable by blasting and comparison with sequences of enteroviruses in GenBank. Among them, 146 cases were CV-A5 (4.56%). The result indicated that CV-A5 emerged as one of the six main serotypes associated with HFMD. All samples were subjected to cell isolation in RD and Vero cells. Among them, 188 strains of enteroviruses belonging to 18 serotypes were isolated in either RD or Vero cells. Four strains were isolated in RD cells and the isolation rate of CV-A5 in RD cells was low (4/146 of CV-A5 positive swabs). Three of four isolates grew at titers higher than 1 × 10^7^ CCID_50_/ml and were also adapted to Vero cells. The complete nucleotide sequences of Vero-adapted isolates were determined for recombination analysis.

### Determination of the complete genome

**Fig. 1:**
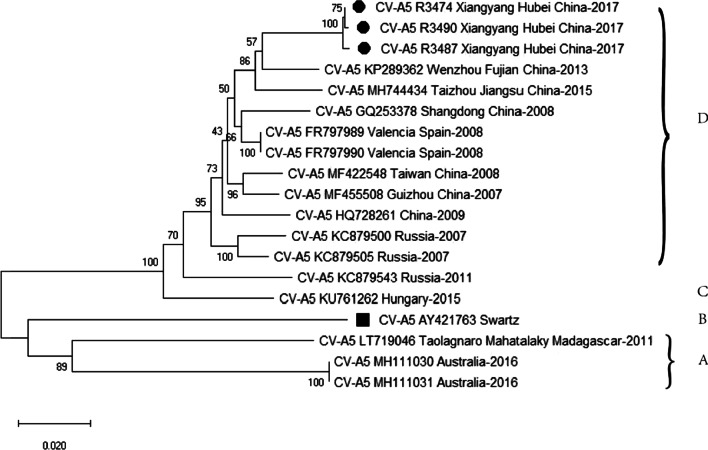
Phylogenetic analysis of Xiangyang CV-A5 isolates and reference strains from Genbank based on the VP1 sequences. *Filled circle* represented the strains isolated from Xiangyang and *Filled square* labeled the CV-A5 prototype Swartz. The GenBank accession no, isolation year and place of each reference strain were indicated. The phylogenetic tree was reconstructed by the neighbor-joining method on the basis of the Kimura (2-parameter) model implemented in MEGA-X. Bootstrap values of 500 was used for evaluation. The genotype demarcation was shown

The complete genome of the three CV-A5 isolates were determined and the homology of nucleotide between each strain were higher than 99.5% by blasting analysis (Fig. 1). The detailed sequence differences between these three CV-A5 strains were showed in Table 3. The genome of the representative strain CV-A5 R3487/XY/CHN/2017 (abbreviated as CV-A5 R3487) is 7356 nucleotides in length. The size of ORF is 6576 bases encoding a polyprotein of 2191 amino acids, which is flanked by the 5′ and 3′ UTR (747 and 81 nucleotides, respectively). The genome is polyadenylated at the terminus of the 3′ UTR. The 5′-terminal of primers (18 nucleotides) designed in this experiment were based on the sequence of CV-A5 prototype Swartz.

**Table 3:** The sequence differences between CV-A5 R3474, CV-A5 R3487 and CV-A5 R3490

	Position (Nt/Aa)	CV-A5-R3474		CV-A5-R3487		CV-A5-R3490	
		Nt	Aa	Nt	Aa	Nt	Aa
VP4	876/43	G	R	A	R	A	R
VP2	1115/54	A	T	A	T	G	A
	1377/141	C	P	A	P	C	P
VP3	1818/33	G	E	A	E	A	E
	2095/125	T	A	T	A	C	A
	2356/212	T	I	C	I	C	I
VP1	2809/123	A	E	A	E	G	E
	2872/144	T	N	A	K	T	N
	2885/149	C	P	T	L	C	P
	3302/288	C	S	T	P	C	S
2A	3420/31	G	L	A	L	G	L
	3670/114	T	L	T	L	C	L
2C	4261/62	C	N	C	N	T	N
	4917/281	T	C	C	C	C	C
3A	5070/3	C	P	T	P	C	P
	5205/48	C	P	T	P	C	P
3C	5397/4	T	L	C	L	C	L
	5774/129	A	G	A	G	G	G
	5868/161	C	H	T	H	C	H
3D	5983/17	G	V	A	I	A	I
	6174/80	T	H	C	H	C	H
	6769/278	T	N	T	N	C	N
	6874/313	G	T	G	T	A	T
	7076/381	C	S	C	S	T	S
	7215/427	A	K	G	K	A	K

### Serotyping and phylogenetic analysis of Xiangyang isolates

Blasting analysis based on the VP1 gene showed that homologies of the CV-A5 strains in Xiangyang with the prototype Swartz were 81.6%-82.0% for nucleotide and 94.9%-95.9% for amino acid, respectively. In contrast, homologies with other serotypes were below 75% for nucleotide and 85% for amino acid, respectively. Based on the criterion of the molecular typing recommended by Oberste [9], Xiangyang isolates were identified as CV-A5 serotype. To determine the phylogenetic relationship between Xiangyang CV-A5 strains and other CV-A5 strains available in the Genbank, a phylogenetic tree was constructed based on the complete VP1 sequence with 16 CV-A5 strains representing genotypes A-D, including six reported Chinese strains (Fig. 1). The phylogenetic dendrogram indicated that the three Xiangyang CV-A5 strains clustered into genotype D and shared identities of 95.9–96.3% and 98.3%-99.3% in nucleotide and amino acid sequences, respectively. Moreover, the Xiangyang isolates displayed the highest similarity with Wenzhou CV-A5 isolate in 2013 [26]. However, there was a clear genetic distinction between the Xiangyang CV-A5 isolates and the prototypes Swartz or other reported CV-A5 isolates elsewhere.

### Recombination analysis

Previously, we reported that a Xiangyang CV-A2 R1580 strain was a recombinant which shared high sequence identity with CV-A5 prototype Swartz in P2 region [27]. To investigate the genetic mechanism of the emerging CV-A5 associated with HFMD epidemic at Xiangyang in 2017, the viral genome regions of CV-A5 R3487 virus, including the 5′ UTR, P1, P2 and P3 regions, were phylogenetically analyzed for genetic relationships with other eight CV-A5 reference sequences.

**Fig. 2:**
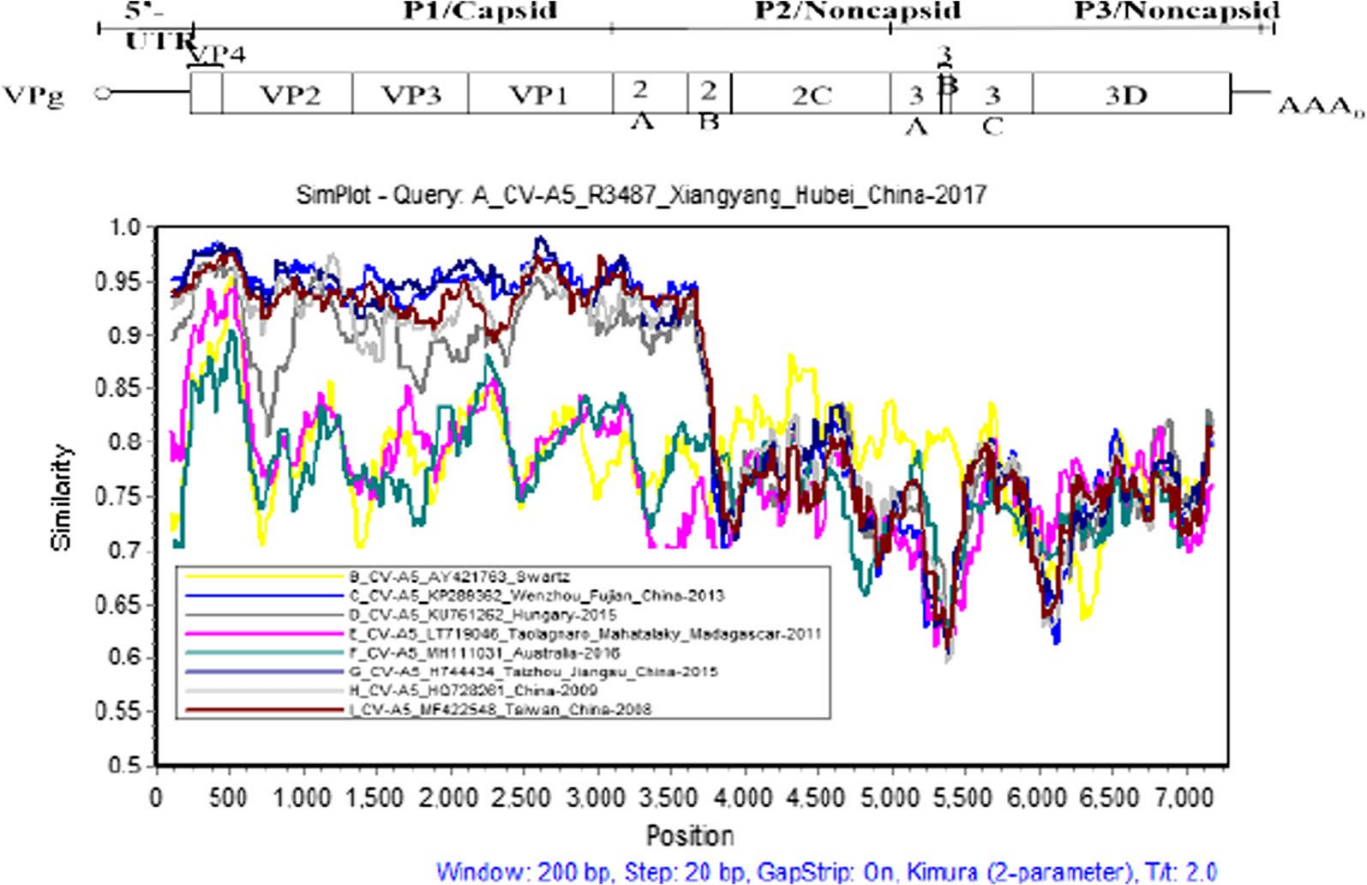
Similarity plot analysis of the whole genome of the Xiangyang CV-A5 R3487 strain. The similarity was calculated in a sliding window size of 200 nucleotides (nt) moving in 20 nt steps, using the Kimura 2-parameter distance method. The sequence of CV-A5 R3487 strain served as a query sequence

**Fig. 3:**
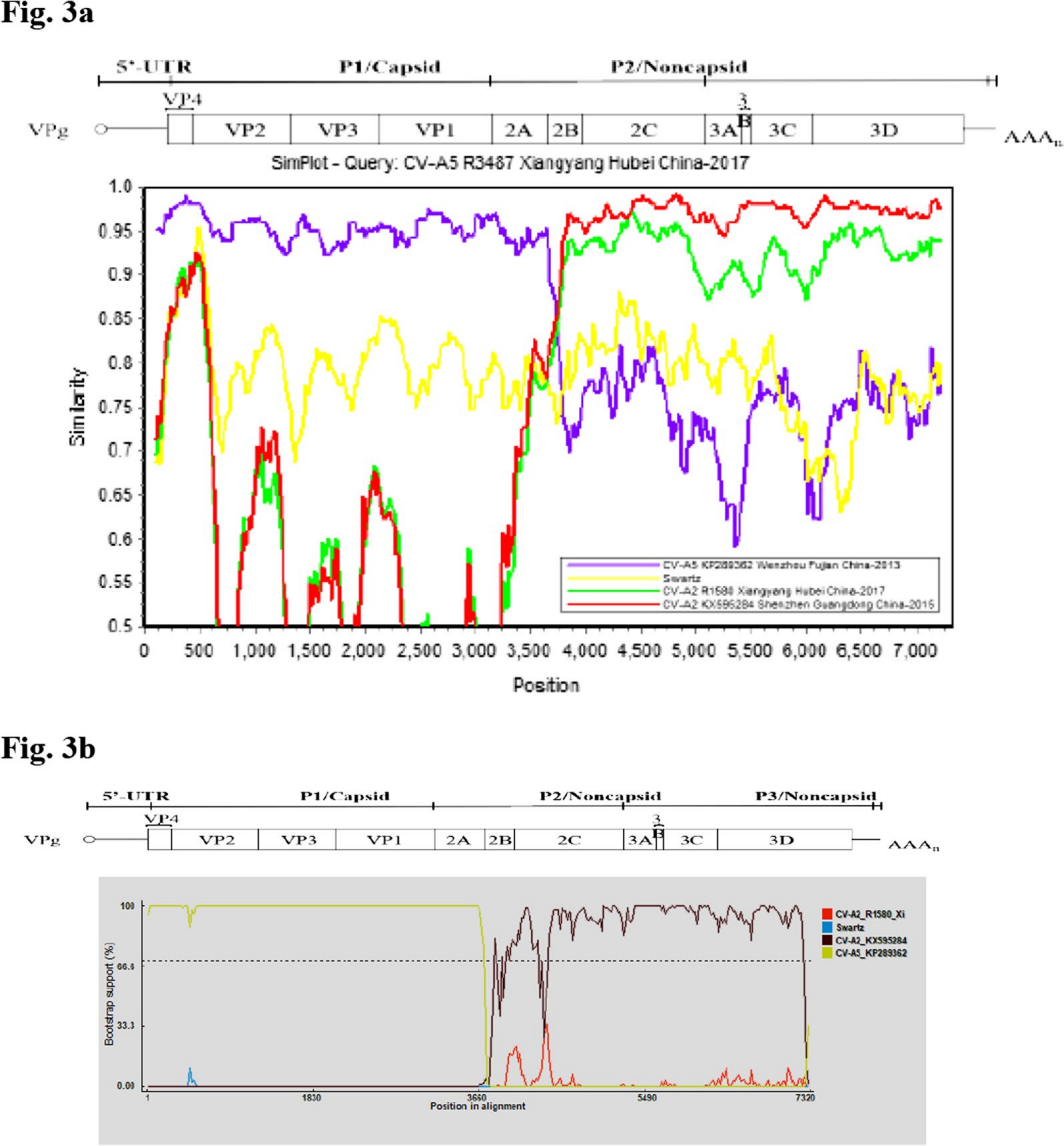
Identification of recombination of Xiangyang CV-A5 R3487 based on the whole genome. Similarity analysis (**a**) and bootscaning analysis (**b**) were performed in a 200 nt sliding window. Each point indicated similarity between the Xiangyang CV-A5 R3487 strain and other EV-A strains in a 20 nt moving step. Kimura (2-parameter) model was used in the analysis. The strains indicated by orange, green, blue and grey were Xiangyang CV-A2 R1580, Shenzhen CV-A2 (KX595284), CV-A5 prototype Swartz and Wenzhou CV-A5 (KP289362) isolates, respectively. The sequence of CV-A5 R3487 strain served as a query sequence

The multi-sequences were scanned by SimPlot software (Version 3.5.1) to generate the similarity plot showed in Fig. 2. Compared with other eight CV-A5 strains, the similarities of CV-A5 R3487 decreased dramatically from the 2B to 3′ UTR region. This result implied that there might be a recombination event with other serotypes in the 2A/2B junction of CV-A5 R3487 strain. As shown in Fig. 3a, in the 2B, 2C, P3 and 3′ UTR regions, CV-A5 R3487 displayed the highest similarity with Shenzhen CV-A2 isolate (KX595284) in 2015 and CV-A2 R1580 strain isolated in Xiangyang in 2017. However, in the 5′ UTR, P1 and 2A regions, the sequence similarities were relatively higher with Wenzhou CV-A5 strain (KP289362) isolated in 2013. Bootscanning analysis was performed, which confirmed a recombination event occurred at the nucleotide (nt) position 3791 in the 2A region of strain CV-A5 R3487 (Fig. 3b). Taken together, these results suggested that a recombinant event may occur between CV-A5 R3487 and CV-A2 originated from an imported (Wenzhou) or a local (Xiangyang) CV-A2 strain.

**Fig. 4:**
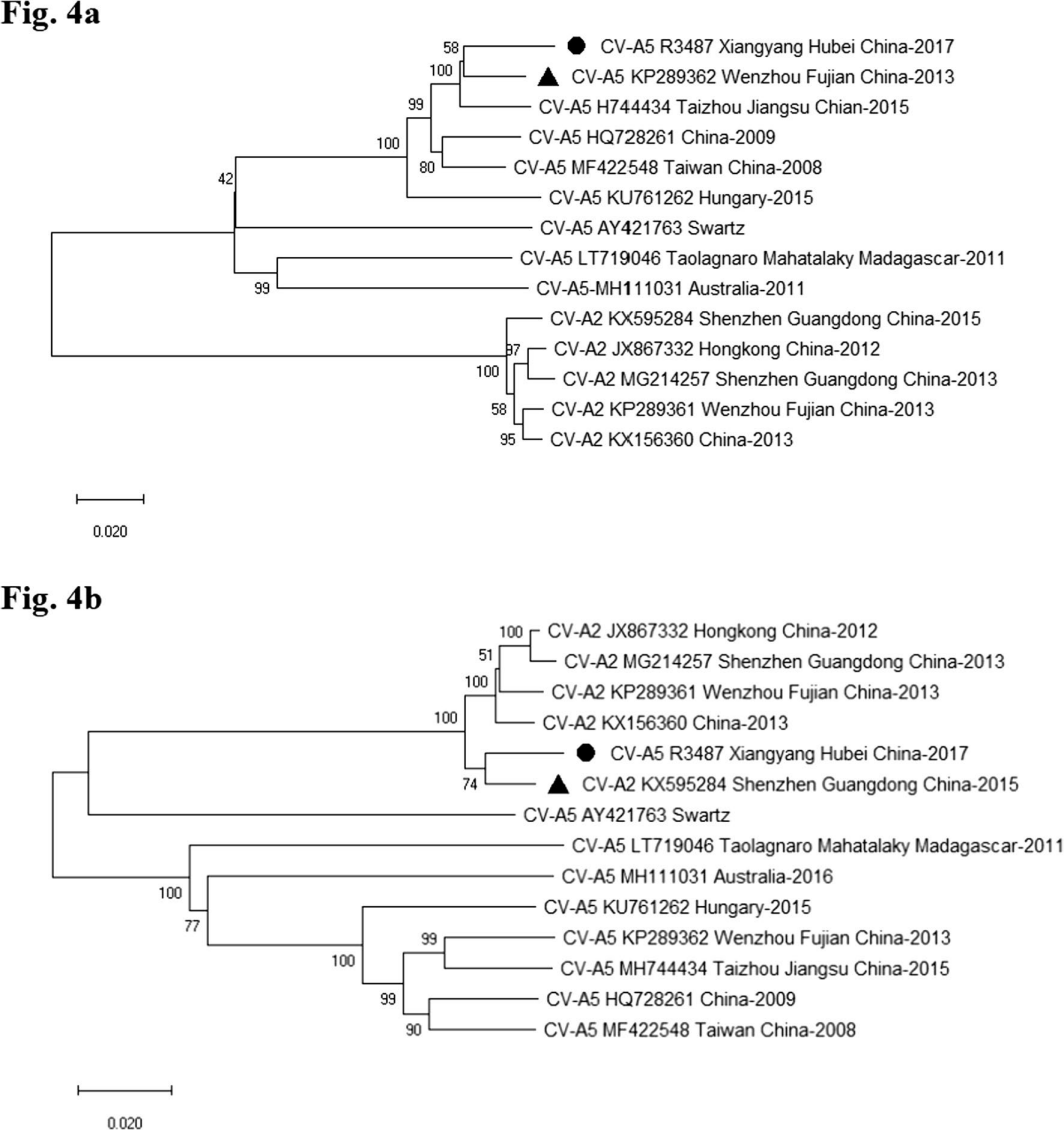
Phylogenetic trees of genetic fragments for Xiangyang CV-A5 R3487 and other CV-A5 and CV-A2 strains, including 5′ UTR-2A (nt 1–3791) (**a**) and 2A-3′ UTR (nt 3792-7404) (**b**) region, were illustrated in figure. Isolate of Xiangyang CV-A5 R3487 was marked with *filled circle*, the strain of Wenzhou CV-A5 (KP289362) or the strain of Shenzhen CV-A2 (KX595284) was marked with *filled triangle* in figure a or b, respectively. The phylogenetic trees were reconstructed using neighbor-joining method with the Kimura (2-parameter) model. Bootstrap values of 500 was used for evaluation

Finally, up- and down-stream regions before and after the breakpoint 3791 were aligned for sequence comparison. The result showed that the 5′-half (nt 1–3791) and 3′-half (nt 3792–7356) were clustered with CV-A5 and CV-A2 strains tested, respectively (Fig. 4a, b). The novel CV-A5 R3487 recombinant obtained the 5′ UTR, P1 and partial 2A regions and downstream partial 2A, 2B, 2C, P3 and 3′ UTR regions of CV-A2 KX595284 strain. A novel CV-A5/CV-A2 recombinant emerged and caused an outbreak of HFMD in Xiangyang in 2017.

## Discussion

RNA viruses exist as quasispecies when they evolve in nature and are passaged in cells or experiment animals [28–30]. The lack of fidelity and proofreading of the RNA-dependent RNA polymerase (RdRp) of these viruses results in high error rates through nucleotide mis-incorporation during genome replication, which could explain how these viruses rapidly mutate and evolve [31, 32]. The emergence, evolution and virulence/epidemics of RNA viruses including enteroviruses are also driven by recombination. The mechanisms involve template switching [33, 34] by the RdRp via an intermediate step of duplicated segments [35] or the replication-independent joining of RNA molecules [36]. It is believed that recombination can correct deleterious mutations and provide advantage for emerging new viruses [37].

Numerous intertypic and intratypic recombination events have been reported mostly in the same enterovirus species within and between serotypes of species A to D. Co-circulation and co-infection of several enteroviruses facilitate the emergence of recombinants. Recombination may act against Muller's Ratchet [38, 39] and as a counterbalancing force against high mutation rates [37] by eradicating deleterious mutations. It may also lead to the combination of advantageous properties from various genomes into a new one such as emergence of drug resistance or even evasion from the immune system.

Emergence of new recombinants is often responsible for the infection, transmission, pathogenesis of these viruses and the consequence may be deadly sometimes. For example, a recombinant CV-A2 caused the deaths of several children in Hong Kong in 2012 [40] and a recombinant of CV-B4 resulted in a fatal case of HFMD in Guangxi in 2012 [41]. Emergence and spread of novel recombinants are often associated with an outbreak of a disease in human population [42]. It is believed that a recombinant of EV-A71 with CV-A16 was one of the reasons behind the outbreak of HFMD epidemic of in Fuyang, China in 2008 [43].

The prevailing EV-A71 is a replication domains donor to other species A enteroviruses, as recently reported research on recombination [44]. CV-A5 also dominated its replication part of the genome to EV-A17 and the recombinant circulated in Eastern Asia [45]. There is evidence that suggests recombination contribute to the switch of dominant serotypes and changes of clinical manifestation [46]. In the outbreak of HFMD in Shanghai in 2012, a recombinant of CV-A6 emerged, which resulted in more generalized skin lesions than normal symptoms induced with non-recombinant CV-A6 [46]. Between 2012 and 2013 in many cities and areas, CV-A6 and CV-A10 have emerged and even became the dominant serotypes [4]. Changes of etiological spectrum may result from mutations and recombination. Following the largescale vaccination of EV-A71 vaccine in China, it is important to closely monitor the new emerging pathogens for disease control measures and for development of multivalent vaccines against HFMD [47].

In Xiangyang, China in 2017, the HFMD outbreak was mainly caused by six main serotypes including CV-A6, A16, A10, A2, A5 and EV-A71. Meanwhile, other 12 serotypes were also detected in clinical samples of HFMD patients at very low proportions. The co-circulation of multiple serotypes increased the chances of co-infection and intertypic recombination [44]. In a period of nine months in 2017, CV-A2 and CV-A5 were co-circulating with case numbers of 117 and 146, respectively. Complete sequence analysis of 3 representative CV-A5 isolates showed that they were recombinants between CV-A5 with a breakpoint at 2A region.

In this study, 8 pairs of primers spanning the recombination hot spots based on published paper [8] were designed for sequence amplification. Unfortunately, by agarose gel electrophoresis, only 7 fragments of 5 samples could be amplified in the right size of PCR products. Interestingly, through blast comparison, we found that 2 of them had the possibility of recombination, CV-A5 R1382 and CV-A5 R1307. The similarity in 2A-2C region between CV-A5 R1382 and CV-A4 (MH086049.1) was 97.95%, and the similarity in 3A-3C region between CV-A5 R1307 and CV-A2 (MN419014.1) was 98.74%. CV-A5 R1382 and CV-A5 R1307, like CV-A5 R3474, CV-A5 R3487, CV-A5 R3490, were also collected in April, 2017. The deficiency of this experiment was that it was difficult to obtain the complete genome of CV-A5 R1382 and CV-A5 R1307. Although one recombination hot spot was determined, further studies should be performed to clarify the potential recombination.

The limitation of this study was that only 8 and 4 cell isolates were obtained for CV-A2 and CV-A5, respectively. The complete genome sequences of all CV-A5 samples were difficult to complete due to degradation of viral RNA. The ratio of parental strains to recombinants was not known. It was not clear whether recombination occurred in or spread to Xiangyang, as the parental donor strain providing P2 and P3 region was not detected. Nonetheless, further study should be performed to investigate the origin, evolution of CV-A5 parental strain and recombinant, and its pathogenicity in mouse model and human. Following the largescale vaccination of EV-A71 vaccine in China, it is worthwhile to closely monitor the new emerging pathogens for disease control and for development of multivalent vaccines against HFMD.

## Conclusions

This study presents complete sequence analysis of 3 representative CV-A5 isolates showed that they were recombinants between CV-A5. This result may explain the emergence of CV-A5 as one of the major pathogens of HFMD epidemics in Xiangyang, China.

## Data Availability

All of the sequence data have been deposited. The datasets generated and/or analysed during the current study are available in the NCBI. The GenBank number of the sequence involved in the manuscript are: MN663160(CV-A5 R3487), OK334537(CV-A5 R374), OK334538(CV-A5 R3490), OK393668(CV-A5 R1307), OK393669(CV-A5 R1382). The datasets used and analyzed during the current study are available from the corresponding author on reasonable request.

## Abbreviations

HFMD: Hand, foot and mouse disease
ORF: Open reading frame
RDP: Recombination detection program
RdRp: RNA dependent RNA polymerase
